# Toward the Patient Participation Pathway: A Mixed Methods Study of Patients With Cancer and Other Chronic Diseases

**DOI:** 10.1002/cnr2.70258

**Published:** 2025-06-30

**Authors:** Paloma Casado Durández, Eleuterio A. Sánchez‐Romero, Alicia Negrón Fraga, Concepción Rey Benayas, Claudia Ruiz‐Huerta García de Viedma, Davinia Medina Ferrer, Ildefonso González Solana, Maria Caballero Nahúm, Mercedes Vinuesa Sebastián, Rosa María de la Salazar Guerra, Nina Cadeau‐Comte, Laura Jiménez‐Ortega, Juan Nicolás Cuenca‐Zaldívar

**Affiliations:** ^1^ Medical Specialist in Clinical Analysis Hospital Universitario del Sureste Madrid Spain; ^2^ Research Group in Nursing and Health Care Puerta de Hierro Health Research Institute‐Segovia de Arana (IDIPHISA) Majadahonda Spain; ^3^ Interdisciplinary Research Group on Musculoskeletal Disorders Madrid Spain; ^4^ Physiotherapy and Orofacial Pain Working Group Sociedad Española de Disfunción Craneomandibular y Dolor Orofacial (SEDCYDO) Madrid Spain; ^5^ Department of Rehabilitation Sciences Florida Gulf Coast University Fort Myers Florida USA; ^6^ Ramón y Cajal University Hospital Madrid Spain; ^7^ Hospital Clínico San Carlos Madrid Spain; ^8^ Hospital Universitario Cruz Roja San José y Santa Adela Madrid Spain; ^9^ University Hospital of Torrevieja Alicante Spain; ^10^ Guadarrama Hospital Madrid Spain; ^11^ University Hospital of La Princesa Madrid Spain; ^12^ Jean Jaurès Toulouse France; ^13^ Department of Psychobiology Complutense University of Madrid Madrid Spain; ^14^ Center of Human Evolution and Behavior UCM‐ISCIII Madrid Spain; ^15^ Psychology and Orofacial Pain Working Group Sociedad Española de Disfunción Craneomandibular y Dolor Orofacial (SEDCYDO) Madrid Spain; ^16^ Universidad de Alcalá, Facultad de Medicina y Ciencias de la Salud Departamento de Enfermería y Fisioterapia, Grupo de Investigación en Fisioterapia y Dolor Alcalá de Henares Spain; ^17^ Primary Health Center “El Abajón” Madrid Spain

**Keywords:** chronic disease, empowerment, patient participation

## Abstract

**Introduction:**

Chronic diseases such as breast cancer, colon cancer, chronic obstructive pulmonary disease (COPD), stroke, diabetes, depression, and myocardial infarction remain leading causes of death worldwide, contributing significantly to premature mortality. Understanding psychosocial impact is essential for comprehensive care. This study aimed to explore how patients with different chronic disease profiles experience participation during the chronic phase, using a mixed‐methods approach.

**Materials and Methods:**

A convergent parallel mixed‐methods design was used to integrate qualitative data from focus group discussions and quantitative data from physical performance tests. A total of 117 patients were recruited through clinical referrals and patient associations, using purposive and snowball sampling. Thematic analysis was performed collaboratively to ensure consistency. Statistical analysis (R v4.1.3) examined the coding and sentiment differences across disease profiles.

**Results:**

The sample included 117 patients (mean age = 58.66 years; majority were female). Significant differences in qualitative coding were found between pathologies (*p* < 0.001), particularly regarding quality of life and emotional experience in COPD and stroke patients compared to others (*p* < 0.05). Topic modeling identified seven relevant topics, and two main coding clusters emerged: one focused on pain and basic activities, and the other on environment and advanced activities. Positive emotions were predominant (*p* < 0.001). The thematic analysis revealed three main themes: quality of life, emotional experience, and empowerment.

**Conclusion:**

Patients with different chronic conditions experienced disease and unique participation. Patients with breast cancer prioritized pain and daily function, while patients with COPD focused on autonomy in self‐management. These findings support the need for individualized disease‐specific approaches to promote meaningful patient participation.

## 1 Introduction

Colon cancer, breast cancer, chronic obstructive pulmonary disease (COPD), stroke, diabetes, depression, and myocardial infarction are among the conditions that contribute significantly to global disability and premature mortality. According to the Global Burden of Disease Study 2019, the worldwide prevalence of these chronic diseases continues to increase, posing multifaceted challenges for healthcare systems [1]. In 2020 alone, more than 19 million new cancer cases were reported worldwide, with colon and breast cancers ranking among the most common diagnoses [2]. In Europe, the incidence and mortality patterns vary notably across regions, reflecting different risk factor profiles and healthcare structures [3, 4]. While cancer represents a dominant concern, stroke survivors often experience considerable emotional distress, particularly when depression emerges as a frequent comorbidity that complicates recovery [5].

Successful adaptation to chronic diagnosis requires effective coping and stress appraisal mechanisms [6, 7]. Critical life events, such as the onset or recurrence of severe illness, can trigger profound psychosocial distress, especially when clinical outcomes remain uncertain [8, 9]. Furthermore, mounting evidence suggests that the presence of earlier adverse experiences or multiple comorbidities may shape resilience or vulnerability upon encountering new health challenges [10, 11]. Structural and societal factors, including discrimination, can also complicate these processes in specific populations [12]. Given these multidimensional influences, the World Health Organization's ICD‐11 classification underscores the importance of conceptualizing chronic diseases as complex biopsychosocial phenomena [13]. Contemporary models of illness representation reinforce this perspective, highlighting how individuals' beliefs and perceptions influence adherence behaviors and overall well‐being [14].

Nevertheless, while medical protocols have advanced, the emotional burden and empowerment processes associated with chronic diseases remain comparatively underexplored [15]. For example, breast cancer survivors frequently report unmet psychosocial needs even after treatment [15], whereas patients with traumatic brain injuries or similar debilitating conditions encounter broad emotional and logistical challenges [16]. Early discharge hospital‐at‐home initiatives can offer valuable benefits but may inadvertently introduce new stressors to vulnerable patients [17]. To address these gaps, recent studies emphasize that interventions that strengthen patient engagement and resilience can substantially improve health outcomes [18]. Specifically, patient‐centered medical homes tailored to chronic diseases such as diabetes or heart disease have demonstrated promising results in enhancing self‐management and psychosocial well‐being [18].

Qualitative methods allow for richer insights into these experiences by capturing patients' personal narratives and motivations [19, 20]. However, maximizing the depth and validity of such inquiries often necessitates mixed methods research (MMR), wherein qualitative findings are integrated with quantitative measures to provide a robust evidence base [21]. This approach is in accordance with emerging guidance that underscores the synergy between rigorous qualitative inquiry and clinical research design [22]. Consequently, the present study employed a convergent parallel MMR design to investigate how patients with colon cancer, breast cancer, and other chronic diseases such as COPD, stroke, and diabetes experience their illnesses emotionally. By analyzing experiences of patients with cancer specifically, we aim to contribute actionable insights into how cancer survivors adapt emotionally and functionally during the chronic phase. Understanding these patterns can guide the development of psychosocial interventions and patient‐centered care pathways tailored to cancer survivors, especially in the context of long‐term quality of life and empowerment.

## 2 Materials and Methods

### 2.1 Study Design

A retrospective study of archived samples using a mixed methods research study [21] was conducted according to the Standards for Qualitative Research Reporting (SRQR) [22] checklist in 117 patients with colon cancer, breast cancer, COPD, stroke, diabetes, depression, and myocardial infarction. The focus groups were conducted at the Hospital Clínico de San Carlos in Madrid and at the Hospital Virgen del Rocío in Seville (Spain) between November 16 and December 14, 2019. Recruitment was conducted through medical consultations at both the hospitals and patient associations. Data for the study were retrieved on December 2, 2023, preserving the anonymity of the individual participants during and after data collection. The procedures were conducted following the Consolidated Criteria for Reporting Qualitative Research (COREQ) statement and checklist [23]. In addition to the Declaration of Helsinki, written informed consent was obtained from all patients for the publication of this study. Although focus group discussions were originally conducted prospectively in 2019 at Hospital Clínico San Carlos as part of routine patient engagement initiatives, the current study represents a retrospective analysis of archived qualitative data in line with the new research objectives. For this reason, the study was submitted for ethical approval to the Local Ethics Committee of the European University of Madrid (Madrid, Spain; CI/2023‐326), which served as the coordinating institution. At the time of submission, the ethics committees of the data collection sites did not oversee secondary research using anonymized data. Therefore, approval by the European University of Madrid was considered appropriate and sufficient for the present analysis.

### 2.2 Setting and Sample

In qualitative studies, sampling aims to select participants who can provide relevant information regarding the phenomenon being studied. In this study, we used a purposive non‐probabilistic sampling approach [24] to deliberately select subjects relevant to the research question. This was followed by a snowball technique in which participants referred to others in similar circumstances who met the inclusion criteria [25]. This approach avoids the bias derived from the snowball technique [26] and is a well‐known method used in qualitative research [27].

In qualitative research, there is no formula to calculate the sample size because the results are not generalizable [25]. In this study, the recommendations proposed by Hennink et al. [28] were followed, in which 2–4 focus groups were proposed to reach 80% code saturation. However, in the case of depression and colon cancer, it was possible to create a group that allowed 60% saturation. However, the use of explicit and concrete deductive codes and their definitions (see Supporting Information for further details) established a priori requires smaller samples [28].

The inclusion criteria were as follows: (a) over 18 years of age; (b) diagnosed with colon cancer, breast cancer, COPD, depression, stroke, diabetes, or myocardial infarction; and (c) in the chronic phase or discharged from medical care. The exclusion criteria were as follows: (a) patients with cognitive or communication disorders, and (b) patients in the acute or subacute phase.

### 2.3 Procedure and Data Collection

The data used in qualitative research came from different sources, and in the present study, they were collected from groups of subjects representative of each target population (i.e., cancer, myocardial infarction, etc.) who discussed the topic of interest [25]. The moderator directed this debate based on the research objectives reflected in a guide with the topics to be discussed to guarantee the legitimacy of the results and reduce biases in the discussion [21, 29].

The study was based on the phenomenological framework [27], as the aim was to understand the life experiences involved in the disease, specifically in aspects related to quality of life, emotional experience, and empowerment [30]. To this end, the codes and categories to be investigated were established a priori in a document agreed upon by members of the research team [31] (Table S1).

The moderators of each group structured their development according to the following guidelines: (a) presentation in which participation is encouraged, the moderator introduced the session and informed that it would be recorded, then the participants were encouraged to actively participate; (b) the moderator posed each of the three research questions in an orderly manner (experience, impact on quality of life, and empowerment), allowing free interaction of the participants and intervening only to redirect the discourse if it deviated excessively from the planned questions; and (c) thanked again for the participation and closed the group.

Twelve focus group discussions (FGDs) were conducted and distributed across six disease profiles. Each group included six to ten participants and lasted approximately 60 min. The sessions were moderated by two experienced researchers trained in qualitative methods and communication in healthcare contexts. Both moderators held postgraduate qualifications in qualitative research methodology and conducted prior FGDs on clinical populations.

Transcripts from each group were used as the primary tools for data collection. Group interactions were audio‐recorded and transcribed verbatim. In total, 1020 min of data collection was recorded, with an average of 60 min per group.

### 2.4 Data Analysis

A deductive thematic analysis was conducted using predefined codes established by the research team [32]. The process began with repeated readings of the transcripts to identify the relevant meaning units. Two researchers independently coded the data, followed by iterative comparisons and discussions to resolve discrepancies. The codes were then grouped into conceptually related categories, from which overarching themes were developed through team consensus. This procedure was repeated across multiple coding cycles until thematic saturation was achieved. The analysis was guided by a structured framework to ensure coherence and consistency with the study objectives.

#### 2.4.1 Methodological Rigor

Methodological rigor in the qualitative phase of the study was ensured through the application of Guba et al.'s criteria [33], which guided the conduct and interpretation of focus group discussions by addressing credibility, transferability, dependability, and confirmability (Table S2).

### 2.5 Ethical Considerations and Data Protection

In‐depth interviews were conducted with the participants' anonymity. Confidence was ensured through strict adherence to ethical guidelines. All participants were assigned pseudonyms during the transcription process, and no identifying information was included in the final dataset. The video recordings were stored securely and were only accessible to authorized research team members. After transcription and verification of data accuracy, the video files were permanently deleted. Furthermore, the transcribed data were anonymized by removing any indirect identifiers, ensuring that individual participants could not be traced. The anonymized datasets were used solely for this study and were kept securely on password‐protected servers that were accessible only to the researchers directly involved in the analysis.

### 2.6 Statistical Analysis

#### 2.6.1 Statistical and Cluster Analysis of Qualitative Codes

Statistical analyses were performed using R, version 4.1.3. Differences in qualitative coding frequencies across pathologies were assessed using Fisher's exact test with Bonferroni‐adjusted post hoc comparisons. Effect sizes were calculated using Cramer's V and interpreted as small (< 0.15), moderate (0.15–0.25), or large (> 0.25). Hierarchical cluster analysis was applied to identify groupings among the coded categories. The number of clusters was predefined based on the coding structure. The goal was to triangulate the qualitative categorization with cluster‐derived structures, thus enhancing the robustness of the findings [34].

#### 2.6.2 Topic Modeling and Sentiment Analysis

The transcripts were lemmatized prior to quantitative text analysis. Topic modeling was performed using Latent Dirichlet Allocation (LDA) with Gibbs sampling to detect topic patterns. The optimal number of topics was determined based on log‐likelihood, semantic coherence, and regression coefficients (*R* ^2^) following the criteria proposed by Arun et al., Cao et al., Deveaud et al., and Griffiths et al. [35, 36, 37, 38]. Additionally, Reinert's double‐clustering method was used to explore the latent textual structures [39].

A sentiment analysis was conducted using the Bing [40], Afinn [41], and NRC [42] lexicons. These dictionaries classify Spanish‐language unigrams into positive/negative valence and, in the case of NRC, into discrete emotional categories (e.g., joy, fear, and sadness). Polarity analysis incorporated SO Dictionaries V1.11Spa [43, 44] and Vilares et al.'s negation/amplifier rules [45]. Differences in sentiment across pathologies were assessed using Fisher's exact test and Kruskal–Wallis H test, with the effect size calculated using Cramer's V and epsilon squared (ϵ^2^), respectively.

## 3 Results

A total of 117 patients participated in the study, with a higher proportion of women (55.56%) and an average age of 58.66 ± 9.54 years (Table 1).

**TABLE 1:** Baseline data.

		Overall	Breast cancer	Colon cancer	COPD	Diabetes	Mental health	Myocardial infarction	Stroke
*n*		117	23	9	22	18	16	17	12
Gender *n* (%)	Female	65 (55.56)	23 (100.00)	4 (44.44)	7 (31.82)	11 (61.11)	11 (68.75)	6 (35.29)	3 (25.00)
	Male	52 (44.44)	0 (0.00)	5 (55.56)	15 (68.18)	7 (38.89)	5 (31.25)	11 (64.71)	9 (75.00)
City, *n* (%)	Madrid	49 (41.88)	9 (39.13)	6 (66.67)	10 (45.45)	6 (33.33)	9 (56.25)	8 (47.06)	1 (8.33)
	Sevilla	68 (58.12)	14 (60.87)	3 (33.33)	12 (54.55)	12 (66.67)	7 (43.75)	9 (52.94)	11 (91.67)
Age		58.66 ± 9.54	55.17 ± 2.37	69.00 ± 1.73	65.05 ± 2.77	40.11 ± 2.17	59.94 ± 2.35	60.06 ± 2.11	70.00 ± 2.52

*Note:* Data expressed with absolute and relative values (%).

### 3.1 Qualitative Coding Analysis

Table of codes and category chart and trustworthiness techniques are provided in Tables S2 and S3.

The qualitative analysis allowed the identification of three fundamental themes.

#### 3.1.1 Theme 1: Quality of Life

This theme emerged strongly across all conditions, reflecting the patients' concerns about maintaining basic and instrumental daily activities, social roles, and independence. For example, a COPD patient stated: “Sometimes, just walking to the kitchen is a battle.”

In this category, patients describe the impact of the disease on their daily lives and how it interferes with their social participation. Dependency to satisfy the most basic needs reduces self‐esteem and is the main problem mentioned: “I remember that on the second day they let me get up to go to the bathroom because they left me the bottle to pee and I said no, that wasn't it” (GF2 [11831:11982]). On the other hand, the consequences make it difficult or impossible to carry out leisure activities: “It has changed my life completely. I had a little field, my garden, my fruit trees, I have already put it up for sale. I can't do anything anymore” (GF2 [6652:6969]) and generate the feeling of not being necessary to society: “After the first stroke I remember having my normal life, working in a company, then the second stroke reminds me of working and after the third stroke, a year without working and then early retirement” (GF2 [7059:7263]). Pain constitutes a factor of anticipatory fear, especially in patients with breast cancer, and non‐appearance constitutes a factor of relief: “And when I came out, I woke up without any pain, nothing normal, I got out of bed and sat down.” (GF2 [36977:37073]).

#### 3.1.2 Emotional Experience

Participants often described emotional fluctuations including fear, sadness, anxiety, and moments of hope. These feelings are linked to disease perception, interactions with healthcare professionals, and support networks.

Illness and disability represent vital situations in which the person is more vulnerable to the different processes related to the disease, both due to the treatment received by professionals: “I would like them to treat all patients the same, I felt very well treated” (GF1 COPD [3642:3724]), and by the physical environment itself in which the therapeutic procedures are performed and which are often not “friendly”: “the patient and his family are totally helpless and very hostile, the hospital is very hostile” (GF1 [29642:29746]). On numerous occasions, the illness itself is perceived as vital before and after: “I was reborn on February 9, 2014” (GF1 [7616:7662]) in which patients feel misunderstood even by their own family environment: “They tell you and you, what problem do you have? If they ask you what problem you have and you say it's depression, they tell you to go out, it's your business because they see you well on the outside” (GF1 [1599:1805]).

#### 3.1.3 Empowerment

Some patients, especially those with prior healthcare exposure, reported increased self‐awareness and active participation in decision‐making. Empowerment is associated with disease understanding, goal‐setting, and interaction quality.

Vulnerability and dependence cause loss of control and decision over the disease and globally over any vital situation. Receiving adequate and understandable information, free of technicalities: “It was very technical and very brief in words, I don't know, I didn't give it importance” (GF2 [4031:4103]) allows patients to understand better their process: “I'm learning a lot of things now at the talks I go to. After 8 years I am learning many things” (GF2 [19133:19259]). This understanding of the disease can improve adherence to treatments, especially those related to self‐care: “At diagnosis, my doctor presented me with inhalers and taught me how to use them” (GF1 [3283:3361]), and with the acquisition of healthy habits: “I started doing sports (going up and down stairs)” (GF1 [4920:5016]), “Then I stopped smoking and the cough went away” (GF1 [5484:5525]).

No formal subthemes were identified during the coding process, as the analysis focused on high‐level categories that aligned with the study's research objectives.

#### 3.1.4 Quantitative Exploration of Qualitative Codes

There were significant differences in qualitative coding between pathologies in all categories (*p* < 0.001) with large effect sizes, except for the emotional experience category, in which the effect was small (Table 2). In terms of quality of life, differences occur between the pathologies of breast cancer versus COPD, Breast cancer versus Stroke, COPD versus Diabetes, COPD versus myocardial infarction, and COPD versus stroke. On the one hand, patients breast cancer patients' main concerns are related to pain and a deficit in advanced activities of daily living, compared to COPD patients who complain more of a deficit in basic and instrumental activities and stroke patients with a deficit in instrumental and advanced activities. On the other hand, COPD patients are more concerned about deficits in basic activities than those with diabetes, myocardial infarction, or stroke, who are more concerned about deficits in instrumental and advanced activities. In the emotional experience, differences occurred between the pathologies of breast cancer versus COPD, Breast cancer versus Diabetes, Breast cancer versus myocardial infarction, breast cancer versus Stroke, COPD versus Diabetes, COPD versus mental health, COPD versus myocardial infarction, and COPD versus stroke. On one hand, breast cancer patients worry about the care received and the perception of the disease compared to COPD patients, diabetes, myocardial infarction, and stroke, who report greater concern about the perception of the disease. On the other hand, COPD patients worry more about the perception of the disease than do patients with diabetes, mental health, myocardial infarction, and stroke, who are concerned about both the perception of the disease and the care received. In aspects related to empowerment, differences are seen between the pathologies COPD versus mental health, COPD versus stroke, diabetes versus mental health, and diabetes versus stroke in patients with COPD or diabetes; the main concern is self‐management of the disease compared to patients with mental health or stroke who demand more information (Table S3).

**TABLE 2:** Qualitative codes by focus group.

Category	Code	Breast cancer	Colon cancer	COPD	Diabetes	Mental health	Myocardial infarction	Stroke	*p* ^a^	Cramer's V (effect size)
Quality of life	Advanced activities of daily living (%)	71.4	60.0	31.1	81.8	50.0	66.7	49.1	< 0.001	0.405 (large effect size)
	Basic activities of daily living (%)	0.0	40.0	39.2	6.1	0.0	5.6	3.6		
	Instrumental activities of daily life (%)	0.0	0.0	29.7	12.1	50.0	22.2	47.3		
	Pain (%)	28.6	0.0	0.0	0.0	0.0	5.6	0.0		
Emotional experience	Attention received (%)	38.2	32.4	15.5	43.2	48.1	35.4	35.2	< 0.001	0.144 (small effect size)
	Physical environment (%)	7.9	5.4	7.0	1.7	0.0	0.0	0.8		
	Perception of the disease (%)	38.2	45.9	51.9	41.2	30.8	51.9	40.6		
	Family‐social relationship (%)	15.7	16.2	25.6	13.8	21.2	12.7	23.4		
Empowerment	Self‐management of the disease (%)	44.9	56.2	63.3	62.1	27.8	45.2	33.3	< 0.001	0.253 (moderate effect size)
	Information received (%)	55.1	43.8	36.7	37.9	72.2	54.8	66.7		

*Note:* Data expressed as relative frequencies (%). Cramer's V values are interpreted according to standard thresholds: small (< 0.15), moderate (0.15–0.25), and large (> 0.25). 95% CI: confidence interval.

^a^ Statistically significant at *p* < 0.05 (values shown in red).

**FIGURE 1:**
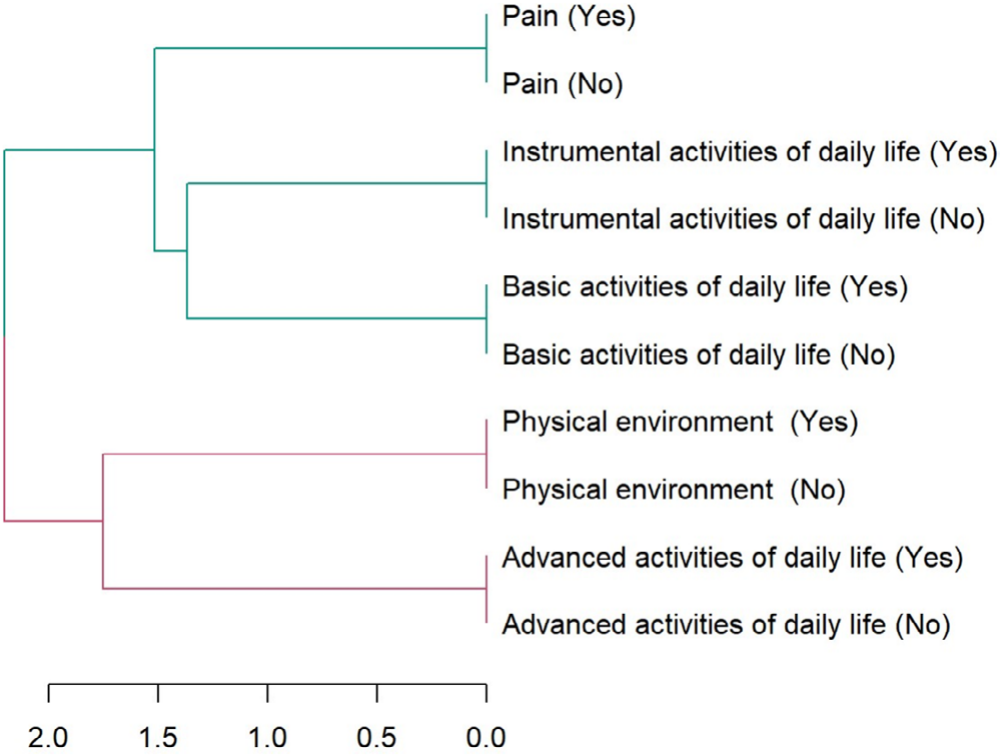
Codes dendrogram.

For qualitative coding, cluster analyses identified two 2 clusters and data stabilization in the third cluster, as indicated by the criterion variation figure (Figure S1). Altogether, the dendrogram shows two large groupings of codes, one with pain and its relationship with the performance of basic and instrumental activities of daily life, and another that relates the physical environment to the performance of advanced activities of daily life (Figure 1).

### 3.2 Quantitative Analysis

In the topic model analysis, the best maximization and minimization values were found in the band of 7–11 topics although the best convergence occurred with seven topics (Figure S2). The *R* ^2^ and coherence values were very similar in all models, with minimal differences between the 7 topics model and the best one (0.041 points of difference in the case of *R* ^2^ and 0.011 points of difference in the case of consistency between the best), whereas the model with seven topics presented the lowest log likelihood value with a difference of 1855.666 points over the second‐best model (Table S4).

The model with seven topics is finally selected because it has better maximization and minimization values, *R* ^2^ and coherence that are very similar to the models with 8, 9, 10, and 11 topics and a clearly lower log likelihood. We verified how Topic 1 appears in all groups, assuming the role of the wildcard theme. However, a very different probability of topic assignment was observed between groups: topic 3 appeared preferentially in the breast cancer groups, topics 7 and 6 in the diabetes groups, topic 5 in the COPD groups, topic 2 in the stroke groups, topics 4 and 5 in the myocardial infarction groups, and topic 4 in the mental health group, whereas in the colon cancer group, there was no predominant topic (Figure S3).

**FIGURE 2:**
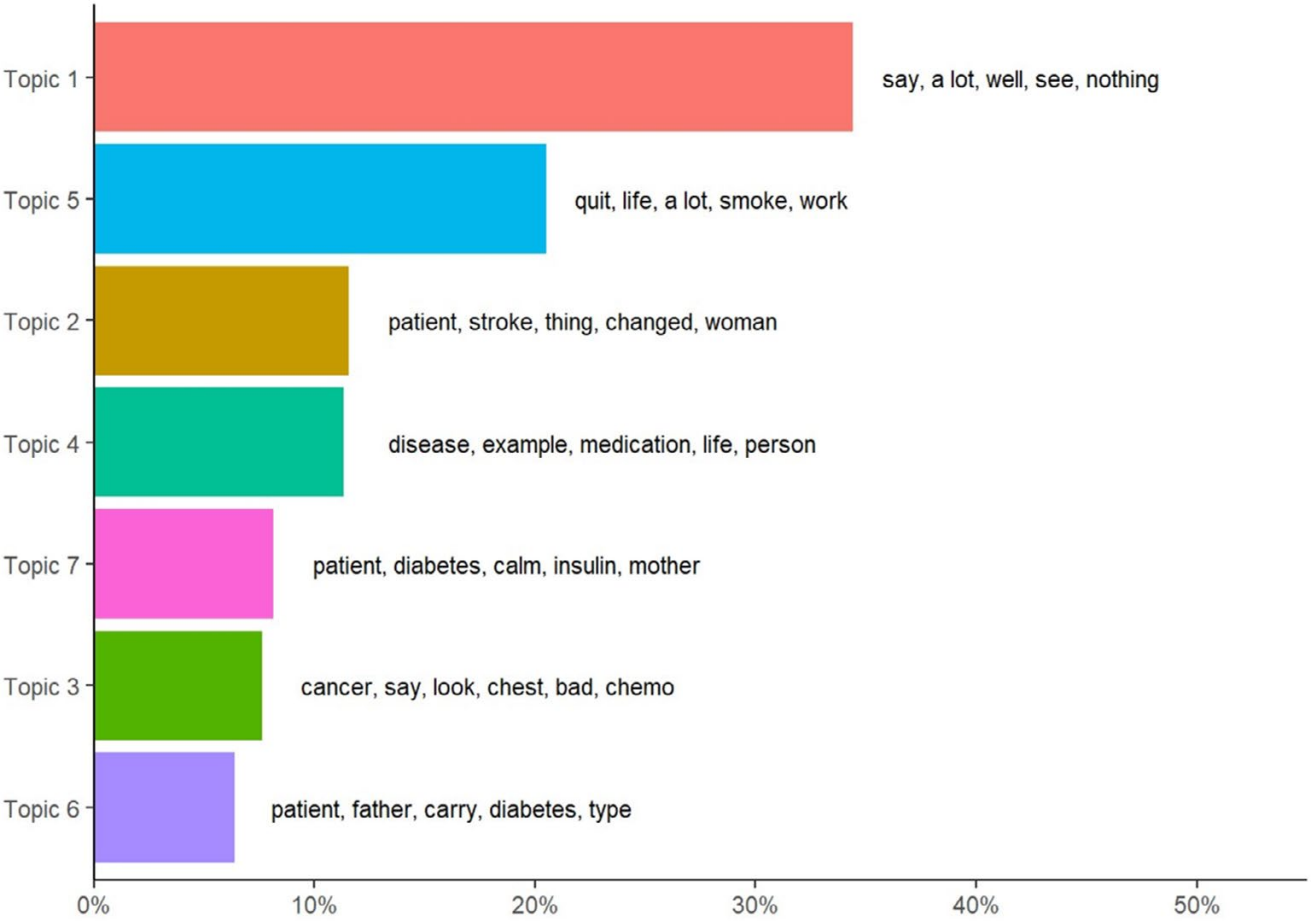
Overall topic weights and top five words.

The topics reflect the main concerns of the groups to which they are preferably assigned; thus, in the breast cancer group, it is represented by topic 3, which focuses on the impact of chemotherapy after cancer detection; in the diabetes group, the topics with the greatest impact are topic 7, referring to problems related to insulin administration, and topic 6, which refers to the impact of the disease and its type on the patient's life; in the COPD group, topic 5 reflects the importance of quitting smoking; in the stroke group, topic 2 with the vital impact of the disease and how it is the partner who takes care of the patient; in the case of the myocardial infarction group, the side effects of the medication, reflected in topic 4, and smoking as a risk factor reflected by theme 5 characterize this process; in the mental health group, again it is the impact of medication and its effects in all areas of life, represented in topic 4, that focuses on concerns (Figure 2).

**FIGURE 3:**
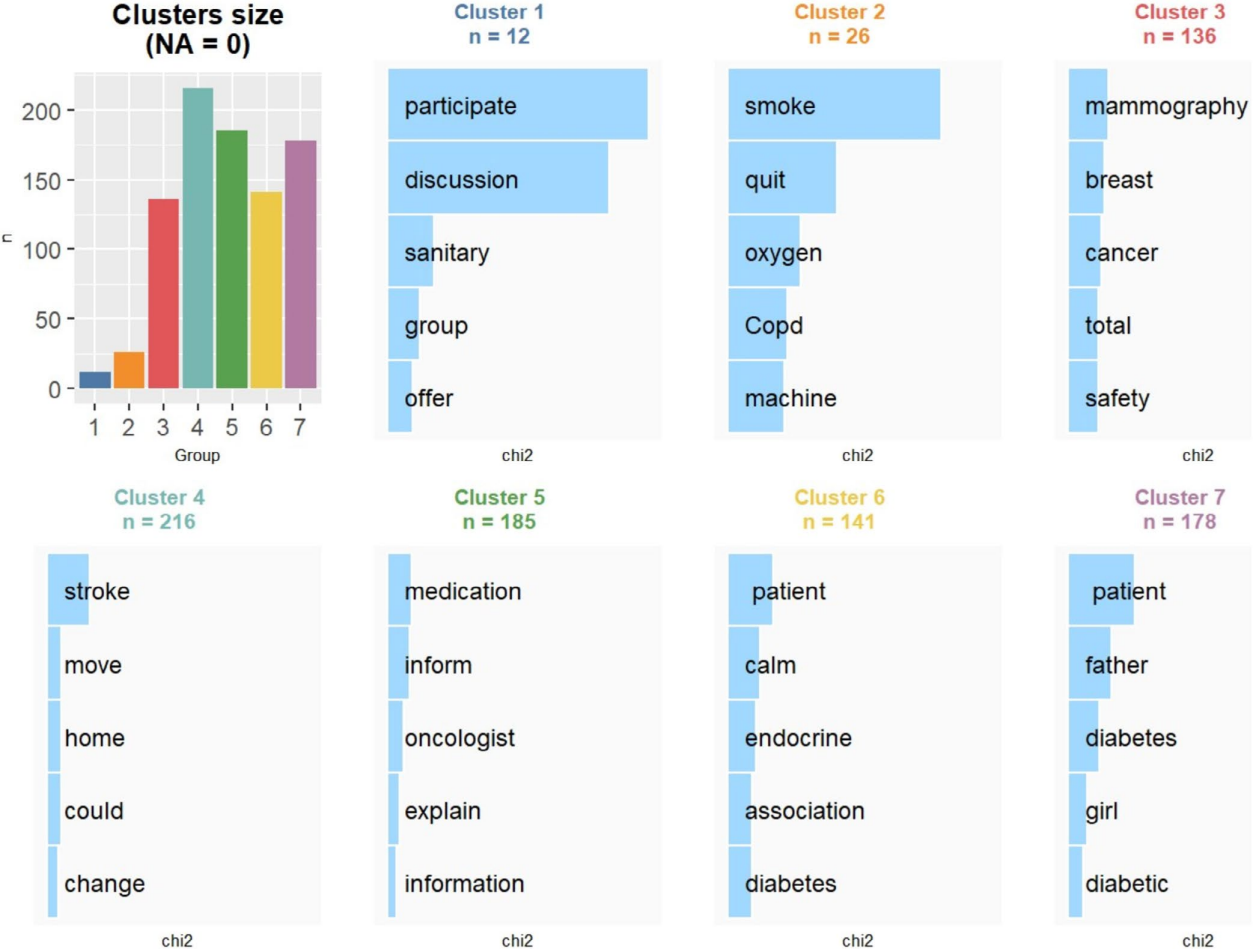
Rainette cluster classification.

Reinert's cluster analysis showed that the most clearly represented groups were the COPD group in Cluster 2, which focused on the importance of quitting smoking and the burden of having to be connected to the oxygen machine; the breast cancer group in Cluster 3, which alludes to the importance of early diagnosis, and 5, which highlights the importance of receiving adequate information, especially that related to medication from the specialist; the diabetes group in Cluster 6, which highlights the importance of support from associations; and 7, which reflects the importance of family support; Cluster 4 characterizes the stroke group, which reflects the problems of the after‐effects upon returning home. Finally, Cluster 1 represents a wildcard grouping related to the greeting and exposition of the groups (Figure 3).

The percentage of textual segments in each group assigned to the clusters corroborates the hypothesized representation in the dendrogram, with the COPD group represented by Cluster 2, the breast cancer group by Clusters 3 and 4, the diabetes group by Clusters 6 and 7, and the stroke group by Cluster 4. Likewise, this indicates that the myocardial infarction and colon cancer groups are fundamentally represented by Clusters 4 and 5, similar to the stroke group, while the mental health group presented a predominance of Cluster 4, indicating that concern about sequelae when returning home is a common theme among these three groups. Finally, Cluster 1 had minimal representation across all groups, which reinforces its wildcard character; the total weight shows how Clusters 4 and 5 are present in the majority of groups (88.235% and 100%, respectively), followed by Clusters 7 and 3 (64.706% and 64.706%, respectively), indicating that the main concerns focus on receiving adequate information, possible consequences when returning home, family support, and early diagnosis (Table S5).

Significant differences in the feelings and emotions expressed between the focus groups were confirmed (*p* < 0.001) with small effect sizes and moderate polarity (Table 3). Systematic differences were observed among all pathologies. With the NCR dictionary, positive feelings are predominant except in myocardial infarction; in the case of the Afinn dictionary, negative feelings are predominant; and with the Bing dictionary, except for diabetes and stroke. The most negative polarity occurred in patients with colon cancer, and the most positive polarity occurred in patients with diabetes (Table S6).

**TABLE 3:** Sentiment and polarity analysis.

		Breast cancer	Colon cancer	COPD	Diabetes	Mental health	Myocardial infarction	Stroke	*p* ^a^	Cramer's V (95% CI)
NCR dictionary sentiments	Negative (%)	46.4	45.6	44.4	27.1	45.3	59.3	33.9	< 0.001	0.192 (0, 1)
	Positive (%)	53.6	54.4	55.6	72.9	54.7	40.7	66.1	NA	
NCR dictionary emotions	Anger (%)	9.1	10.7	7.1	6.0	7.8	7.7	6.5	< 0.001	0.074 (0, 1)
	Anticipation (%)	13.7	19.4	14.7	22.3	12.2	12.7	18.1	NA	
	Disgust (%)	8.4	7.8	7.8	5.6	8.6	7.5	5.7	NA	
	Fear (%)	19.8	20.4	19.3	15.0	23.3	23.6	16.3	NA	
	Joy (%)	6.0	1.9	5.6	7.3	4.3	3.9	7.4	NA	
	Sadness (%)	20.8	22.3	18.2	13.6	19.9	25.1	16.3	NA	
	Surprise (%)	4.7	2.9	7.4	6.0	3.2	6.5	7.2	NA	
	Trust (%)	17.4	14.6	20.1	24.2	20.8	12.9	22.5	NA	
Afinn dictionary sentiments	Negative (%)	62.9	78.3	67.9	49.0	57.1	71.2	52.6	< 0.001	0.144 (0, 1)
	Positive (%)	37.1	21.7	32.1	51.0	42.9	28.8	47.4	NA	
Afinn dictionary scores	−5 (%)	0.4	0.0	0.0	0.3	0.0	0.0	0.2	< 0.001	0.094 (0, 1)
	−4 (%)	0.1	1.7	0.0	0.2	0.7	0.0	0.4	NA	
	−3 (%)	7.8	15.0	10.3	3.4	4.5	5.6	4.2	NA	
	−2 (%)	16.5	23.3	14.7	12.5	26.0	30.7	15.4	NA	
	−1 (%)	38.0	38.3	42.9	32.6	26.0	34.8	32.3	NA	
	1 (%)	25.4	13.3	14.0	36.2	23.5	15.4	32.7	NA	
	2 (%)	5.9	5.0	15.4	9.8	14.0	10.0	9.0	NA	
	3 (%)	5.2	3.3	2.7	4.8	5.4	3.1	5.5	NA	
	4 (%)	0.6	0.0	0.0	0.2	0.0	0.3	0.2	NA	
	5 (%)	0.1	0.0	0.0	0.0	0.0	0.0	0.0	NA	
Bing dictionary sentiments	Negative (%)	58.0	63.0	59.2	34.8	62.5	64.6	42.8	< 0.001	0.22 (0, 1)
	Positive (%)	42.0	37.0	40.8	65.2	37.5	35.4	57.2	NA	
Polarity phrases score		−0.08 ± 0.29	−0.28 ± 0.20	−0.06 ± 0.39	0.31 ± 0.40	−0.15 ± 0.44	−0.14 ± 0.18	0.14 ± 0.37	< 0.001	0.155 (0.109, 0.224)*

*Note:* Data expressed with mean ± standard deviation or with relative values (%); 95% CI: 95% confidence interval; *ϵ2 effect size.

^a^ Significant if *p* < 0.05 (shown in red).

## 4 Discussion

This study employed a phenomenological approach to explore patients' lived experiences of chronic diseases, focusing on emotional responses, quality of life, and empowerment. Thematic and polarity analyses revealed distinct psychosocial profiles across disease types among 117 patients, predominantly women (55.56%) with a mean age of 58.66 ± 9.54 years. Consistent with prior research on chronic illness and emotional adjustment [46], patients with breast cancer, stroke, and COPD showed specific concerns regarding pain, loss of autonomy, and activity limitations. In contrast, differences in empowerment were especially prominent in patients with COPD and diabetes, who reported greater emphasis on self‐management and information needs [47].

Significant differences in qualitative coding were observed across pathologies, with large effect sizes in most categories except for emotional experience, which showed a small effect. Quality of life issues were predominant in patients with breast cancer, COPD, and stroke, with the main concerns linked to pain and deficits in daily functioning. Empowerment differences, however, were most apparent among patients with COPD, diabetes, mental health conditions, and stroke. Each group expressed distinct priorities: patients with breast cancer focused on treatment burden, patients with diabetes highlighted challenges related to insulin, and patients with COPD emphasized autonomy in managing respiratory symptoms.

These findings align with previous qualitative studies showing that chronic disease perceptions differ substantially by pathology and are shaped by disease trajectory, symptom visibility, and impact on daily life [48]. Despite these variations, a common need for support, understanding, and individualized communication has emerged across all groups. Differences in expressed feelings and emotions also stood out, with a predominance of positive sentiments in most groups, except for patients with myocardial infarction and colon cancer.

The general framework of health anxiety, encompassing emotional, cognitive, behavioral, and perceptual aspects, may help explain the recurrent fear of relapse or deterioration experienced by many patients [49]. A more refined understanding of pathology‐specific concerns is essential to develop targeted psychological interventions. For example, in cancer survivors with persistent emotional distress, maladaptive metacognitive beliefs such as excessive worry often explain ongoing emotional challenges [50]. Our qualitative approach sheds light on beliefs, emotions, perceived QoL, and motivational factors. Identifying the psychological patterns associated with specific pathologies may provide tailored psychological support. For instance, the more pronounced expressions of anger and negative emotions in breast and colon cancer patients suggest the potential benefit of emotion‐focused cognitive behavioral therapy [51].

Achieving psychological adjustment in chronic illness requires not only identifying barriers, but also recognizing adaptive factors. De Ridder et al. [52] emphasized that psychological adaptation involves maintaining activity, emotional expression, patient empowerment, active self‐management, and reframing illness in a more positive light. Our findings reflect this framework: Patients demonstrated varying degrees of empowerment and positive emotionality across pathologies. Interestingly, despite being among the most lethal diseases, myocardial infarction and breast cancer patients felt more empowered than mentally ill patients [53, 54]. This underscores the need to challenge stigma and promote realistic, constructive narratives about mental illness to enhance empowerment and quality of life [55].

The observed qualitative differences in emotional responses have strong implications in clinical care. Colgan et al. [56] underscored the role of empowerment, communication, and compassion in reducing distress during procedures, such as awake craniotomies. Their findings resonate with our own: Patient perceptions of control and emotional support were pivotal in shaping emotional responses. Similarly, patient‐centered approaches that enhance perceived control could be particularly beneficial in conditions such as cancer, where emotional distress is heightened.

Furthermore, Travis et al. [57] identified barriers to participation in colorectal cancer screening, including procedural anxiety and discomfort concerns. This aligns with our findings on emotional burdens and emphasizes the value of early, personalized communication and psychological reassurance. Patient anxiety remains a major barrier to care adherence, and addressing it through condition‐specific interventions is crucial.

Sentiment and polarity analyses confirmed the emotional variation across conditions. Patients with colon cancer showed the most negative polarity, possibly reflecting anxiety over disease progression and prognosis. Conversely, patients with diabetes displayed the most positive polarity, likely because of greater perceived control over disease management. These findings highlight the need to tailor psychosocial support to the emotional landscape in each condition.

### 4.1 Limitations and Strengths

This study shares common limitations with qualitative research, such as limited generalizability due to small sample sizes and purposive sampling. Potential biases related to focus group dynamics must also be acknowledged: Dominant participants may overshadow quieter voices, while others may hesitate to disclose sensitive emotions. Additionally, emotional expressions in group settings can be context‐dependent. As noted by Travis et al. [52], anxiety and perceptions of medical procedures are shaped by how information is shared, which may limit the transferability of the findings obtained in group settings.

Despite these limitations, the robustness of the study was enhanced by several strengths. First, the use of a mixed methods design, combining thematic analysis with quantitative sentiment and polarity assessment, allowed for methodological triangulation and increased credibility of the findings. Second, detailed descriptions of the study setting and participant characteristics support the cautious transferability to comparable populations. Finally, the relatively large sample size for a qualitative study enriched the diversity and depth of the emotional experiences captured, allowing for a nuanced understanding of chronic illnesses across different pathologies.

While the study faces inherent limitations of phenomenological methods, including interpretative subjectivity, its strengths—notably, the use of mixed methods, detailed transferability criteria, and a sizable sample—offer a solid foundation for understanding patients' emotional experiences with chronic disease.

### 4.2 Practical Implications

Differences in emotional polarity have significant practical implications for psychological support. Patients with more negative polarity, such as those with colon cancer, may benefit from structured emotional interventions, such as CBT. Conversely, patients with diabetes can benefit from programs that reinforce self‐care and support optimism. Healthcare professionals should adapt emotional and psychosocial support strategies to each condition's psychological profile to ensure holistic care.

### 4.3 Future Directions

Future research should adopt longitudinal designs to track the emotional evolution of cancer survivors over time, as this population often faces distinct psychosocial challenges beyond remission. Participatory action research (PAR) involving cancer patients and oncology professionals can support the co‐design of personalized support interventions. Additionally, identifying resilience factors in breast and colon cancer survivors may offer protective strategies to buffer emotional distress. Tailored psychological support models—such as cognitive‐behavioral therapy or mindfulness‐based stress reduction—should be tested specifically in oncology contexts to improve adaptation and quality of life.

Larger and more diverse samples and the use of both focus groups and individual interviews will improve data richness and generalizability. Additionally, studies should explore the protective factors that foster positive emotional outlooks, despite the burden of illness. Incorporating these strategies will enhance our understanding of emotional experiences and inform patient‐centered care models in the context of chronic diseases.

## 5 Conclusions

This study revealed significant differences in the emotional and participatory experiences of patients with chronic conditions, highlighting the need for tailored psychosocial and clinical care strategies. Patients with colon cancer expressed the most negative emotional polarity, emphasizing the necessity for targeted psychological support such as cognitive‐behavioral therapy. Patients with breast cancer prioritized pain and limitations in advanced daily activities, indicating the value of integrated pain management and supportive care. In contrast, patients with diabetes exhibited a more positive emotional outlook, suggesting a greater sense of control that could be reinforced through educational and peer support programs. COPD and stroke patients reported feelings of helplessness linked to disease progression, underscoring the importance of embedding emotional support into rehabilitation efforts. Distinct emotional profiles were also observed among patients with depression and myocardial infarction, focusing on emotional vulnerability and concerns regarding long‐term treatment effects. Across all conditions, the provision of clear, accessible information and condition‐specific emotional support emerged as critical for patient empowerment. Healthcare professionals should adapt their psychosocial support strategies accordingly and integrate routine emotional assessments into care plans to improve patient outcomes and well‐being.

## Author Contributions

Conceptualization: P.C.D. and J.N.C.‐Z. Methodology: J.N.C.‐Z. and E.A.S.‐R. Software: J.N.C.‐Z. Validation: all authors. Formal analysis: J.N.C.‐Z. Investigation: P.C.D., M.V.S., R.M.d.l.S.G., C.R.‐H.G.d.V., C.R.B., A.N.F., D.M.F., I.G.S., and J.N.C.‐Z. Resources: J.N.C.‐Z. and E.A.S.‐R. Data curation: J.N.C.‐Z. Writing – original draft preparation: J.N.C.‐Z., L.J.‐O., N.C.C., and E.A.S.‐R. Writing – review and editing. J.N.C.‐Z., L.J.‐O., N.C.C., and E.A.S.‐R. Visualization: J.N.C.‐Z. and E.A.S.‐R. Supervision: J.N.C.‐Z., N.C.C., and E.A.S.‐R. Project administration: J.N.C.‐Z. All authors have read and agreed to the published version of the manuscript.

## Ethics Statement

The study was conducted in accordance with the Declaration of Helsinki and approved by the Local Ethics Committee of the European University of Madrid, Madrid, Spain (CI/2023‐326).

## Consent

Informed consent was obtained from all the subjects involved in the study. Written informed consent was obtained from the patients for publication of this paper.

## Conflicts of Interest

The authors declare no conflicts of interest.

## Supporting information


**Data S1.** Supporting Information.

## Data Availability

The data that support the findings of this study are available on request from the corresponding author. The data are not publicly available due to privacy or ethical restrictions.
