# Supplementary material for: Toward the Patient Participation Pathway: A Mixed Methods Study of Patients With Cancer and Other Chronic Diseases
Source: Cancer Rep (Hoboken). 2025 Jun 30;8(7):e70258. doi: 10.1002/cnr2.70258 (PMC12207092; doi:10.1002/cnr2.70258)
Supplement: Supplementary file 1 — Data S1. Supporting Information. [file CNR2-8-e70258-s001.docx]

Supplementary material

**Supplementary material. Table 1:** Codes and categories chart.

| **Categories** | **Categories definition** | **Codes** | **Codes definition** | **Codes examples** |
| --- | --- | --- | --- | --- |
| Quality of life | In this category we explore the impact that the disease has on functionality, execution of tasks or activities (personal independence and autonomy), which will in turn determine the participation or development in social situations and the restrictions or difficulties that the individual finds to participate in them | Advanced activities of daily living | They are related to the values, interests, roles, capacities, intrinsic abilities of the people, in addition to the context that surrounds them. They require more complex cognitive abilities. They include: leisure and free time activities (these are activities that are chosen voluntarily by be satisfactory to the individual), social participation (collective interaction with friends, family, co-workers, patient associations, volunteering), labor activity (physical or intellectual activity that receives remuneration) | In the association we have united a lot. We have an incredible friendship. We make many things together, we help each other a lot |
|  |  | Basic activities of daily living | They are defined as activities directed or oriented toward self-care. They are done daily and are necessary for survival: bath/shower, hygiene and grooming, bladder and intestinal continence, dressing, feeding, mobility, sleep and rest | After the stroke I have not been able to walk alone again, I need help for almost everything, except to eat if they cut my food |
|  |  | Instrumental activities of daily life | They are defined as activities that allow you to interact with the environment in which you live. They have a higher level of cognitive and motor complexity. Some instrumental activities are: take care of and maintain a home, use communication systems, control of your medication, routines to maintain good health, healthy eating, caring for other people or a pet | I have aphasia, and although it is very functional because I can make myself understood, it is difficult for me follow conversations with many people and, for example, understand when I talk on the phone. I communicate better face to face |
|  |  | Pain | With this code we reflect the impact that the perceived intensity and frequency of pain has on quality of life. How pain interferes with your daily life | I have constant pain in my shoulder, which doesn’t even let me sleep, I’m desperate |
| Emotional experience | This concept was born in the 1970s associated with the process of strengthening rights and capabilities of vulnerable people or communities, making them gain confidence and protagonism. Human beings must have access to resources and decisions that affect their lives. lives. An empowered patient is a patient with the ability to decide, satisfy needs and solve problems, with critical thinking and control over your life | Attention received | In this code we will evaluate the perception of patients in their interaction with the professional healthcare of any category. It will reflect how they perceive the treatment (closeness, warmth, empathy, kindness, understanding, support and support, coldness, seriousness, dryness, haste, condescension), the attention received by them (professionalism, availability, information) and the verbal and non-verbal communication. Aspects related to waiting, continuity of care, integration will also be reflected. and coordination of services, management of appointments, accessibility and adapted flexibility to the patient’s needs | My doctor only moved the papers and didn’t look at my face - The results should be given as soon as they are available, life is very bad uncertainty |
|  |  | Physical environment | With this code we evaluate the physical environment in which care occurs. The design seeks the comfort of the patient, takes into account their needs, seeks their safety and if is friendly (signage, summons, living rooms) | I do not understand that there is not an isolated space in the emergency services for oncology patients. Being immunosuppressed you should not wait with people with potentially contagious diseases |
|  |  | Perception of the disease | We reflect expressions related to how they experience their illness, its emotional impact, self-perception, illness awareness. We will also explore whether they have received emotional support during the process, and from whom (professional, peer, expert or informal patient of friends, family, volunteering) | I don’t understand why This has happened if I have always led a healthy life - For me, interacting with other patients was fundamental, I felt understood and that my problems were not unique |
|  |  | Family-social relationship | We reflect expressions related to the impact of the disease on family relationships and social as well as at the work level in relation to the emotions generated in these environments | I didn’t know how to tell my children that I had cancer, or whether to tell my parents, they are very old |
| Empowerment | In this section we will study how the patient has experienced the disease and its evolution and How has it influenced your social and family environment | Self-management of the disease | The patient’s awareness and responsibility in the management of their disease will be explored. If has knowledge, skills and strategies to deal with it and uses them, for example: knows how to control symptoms, warning signs, healthier lifestyle habits for your disease. Their motivation for participation in shared decision making. If you take decisions related to your health according to your beliefs, values, needs, taking into consideration the information provided by the health professional, based on the available scientific evidence. If the patient knows his right to autonomy in the management of his illness and chooses or not to exercise it. In which role do you feel most comfortable, as an active or passive patient | When I arrived at the consultation, the doctor told me that I had to choose whether to treat myself or not. Because the recommendation was not clear, the decision was mine. That to me was horrible |
|  |  | Information received | We will explore whether or not quality and adequate information has been received, whether written, verbal, digital (web, video), by the healthcare professional or by peers | I would have liked them to give me more information. I have been concerned about many things I found out when I came to the association, from my colleagues, and I think it should not be So |

**Supplementary material. Table 2:** Trustworthiness techniques.

| **Criteria** | **Techniques performed and application procedures** |
| --- | --- |
| Credibility | Investigator triangulation: team meetings were performed in which the analyses were compared, and themes were identified |
|  | Member checking: this consisted of asking the participants to confirm the data obtained during the data collection |
| Transferability | In-depth descriptions of the study were performed, providing details of the characteristics of researchers, participants, contexts, sampling strategies, and the data collection and analysis procedures |
| Dependability | Audit by an external researcher: an external researcher assessed the study research protocol, focusing on aspects concerning the methods applied and study design |
| Confirmability | Investigator and analysis triangulation |
|  | Researcher reflexivity was encouraged via the completion of reflexive reports and by describing the rationale for the study |

**Supplementary material.** Table 3: Qualitative codes post hoc tests ^a^p values.

|  | **Quality of life** | **Emotional experience** | **Empowerment** |
| --- | --- | --- | --- |
| Breast cancer : Colon cancer | >0.999 | >0.999 | >0.999 |
| Breast cancer : Copd | 0.002 | <0.001 | 0.307 |
| Breast cancer : Diabetes | 0.523 | 0.014 | 0.157 |
| Breast cancer : Mental health | >0.999 | 0.355 | >0.999 |
| Breast cancer : Myocardial infarction | >0.999 | 0.045 | >0.999 |
| Breast cancer : Stroke | 0.024 | 0.001 | >0.999 |
| Colon cancer : Copd | >0.999 | >0.999 | >0.999 |
| Colon cancer : Diabetes | >0.999 | >0.999 | >0.999 |
| Colon cancer : Mental health | >0.999 | >0.999 | >0.999 |
| Colon cancer : Myocardial infarction | >0.999 | >0.999 | >0.999 |
| Colon cancer : Stroke | 0.521 | >0.999 | >0.999 |
| Copd : Diabetes | <0.001 | <0.001 | >0.999 |
| Copd : Mental health | >0.999 | <0.001 | 0.005 |
| Copd : Myocardial infarction | 0.041 | 0.002 | >0.999 |
| Copd : Stroke | <0.001 | <0.001 | 0.001 |
| Diabetes : Mental health | >0.999 | >0.999 | 0.002 |
| Diabetes : Myocardial infarction | >0.999 | >0.999 | >0.999 |
| Diabetes : Stroke | 0.125 | 0.319 | <0.001 |
| Mental health : Myocardial infarction | >0.999 | >0.999 | >0.999 |
| Mental health : Stroke | >0.999 | >0.999 | >0.999 |
| Myocardial infarction : Stroke | >0.999 | >0.999 | >0.999 |

^a^significant if p<0.05 (shown in red).

**Supplementary material. Figure 1:** Cluster variation of criterion (after consolidation).


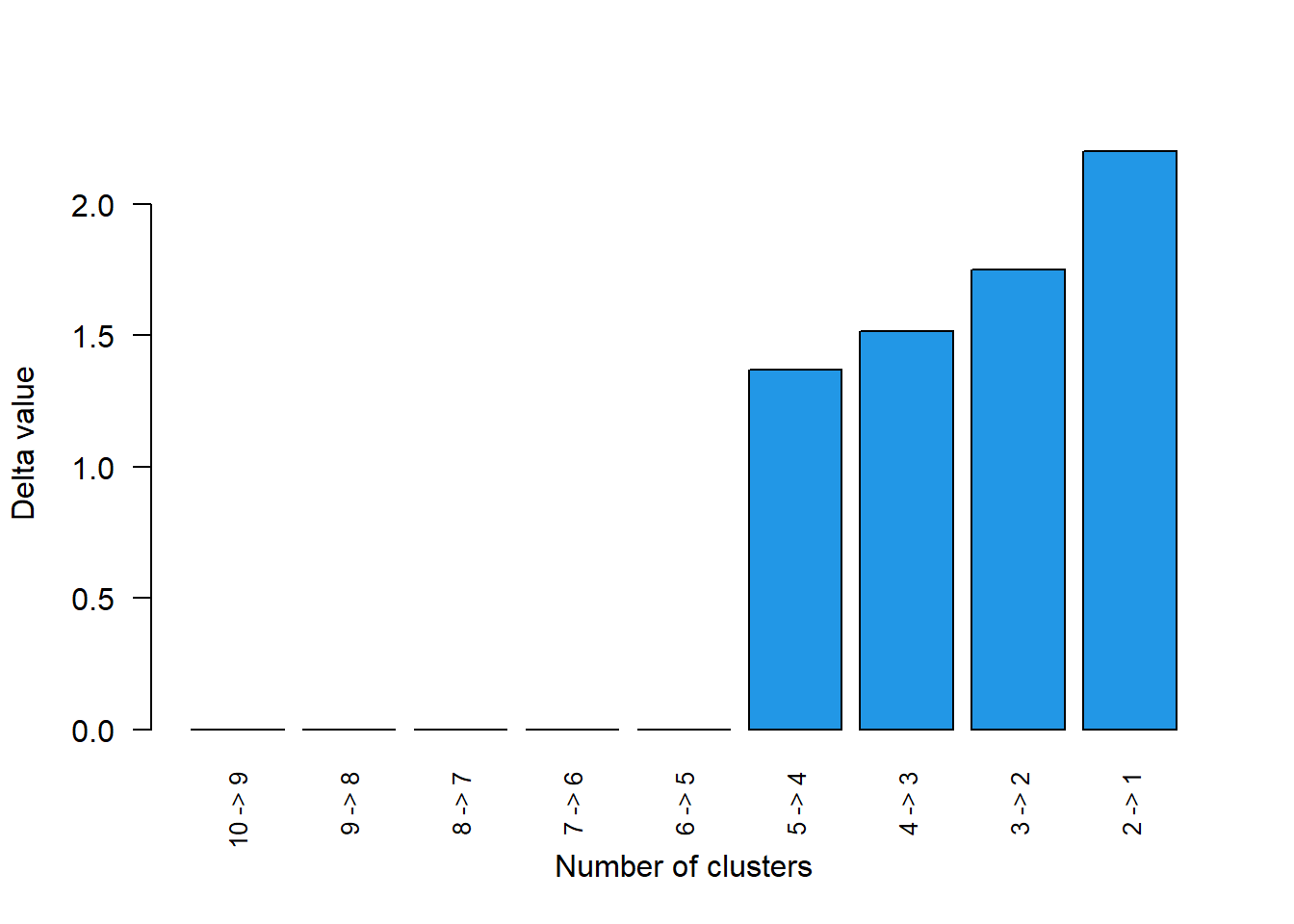


**Supplementary material. Figure 2:** Topics number metrics selection.


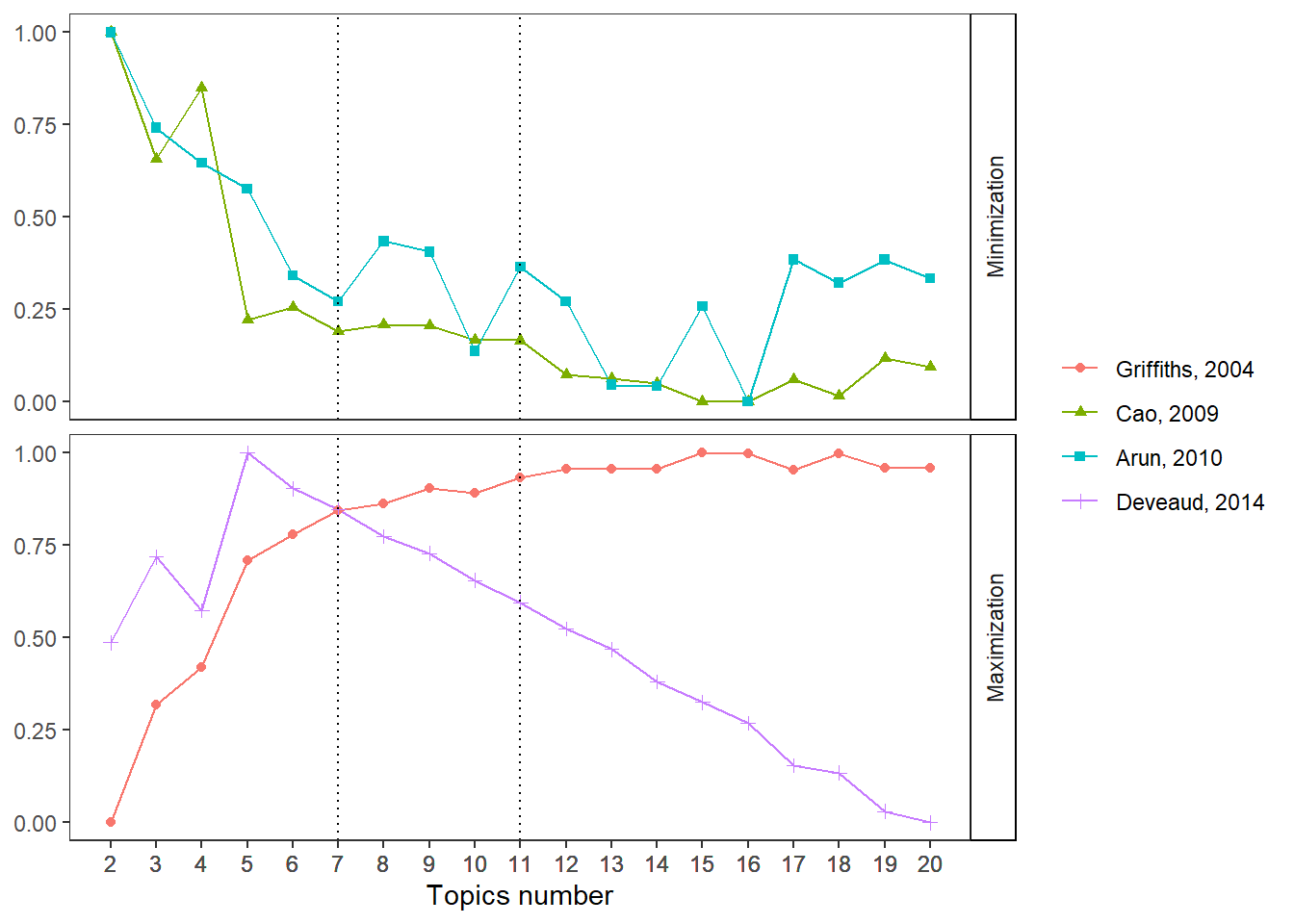


**Supplementary material.** Table 4: Model fit values.

| **Topics number** | **R2** | **Topics number** | **Coherence** | **Topics number** | **Log likelihood** |
| --- | --- | --- | --- | --- | --- |
| 11 | 0.875 | 10 | 0.035 | 7 | -238105.4 |
| 8 | 0.873 | 7 | 0.025 | 8 | -239961.0 |
| 10 | 0.869 | 11 | 0.024 | 9 | -242640.2 |
| 9 | 0.866 | 9 | 0.020 | 10 | -244946.1 |
| 7 | 0.834 | 8 | 0.018 | 11 | -246377.7 |

**Supplementary material. Figure 3:** Gamma (γ) probabilities for each topic within each focus group.


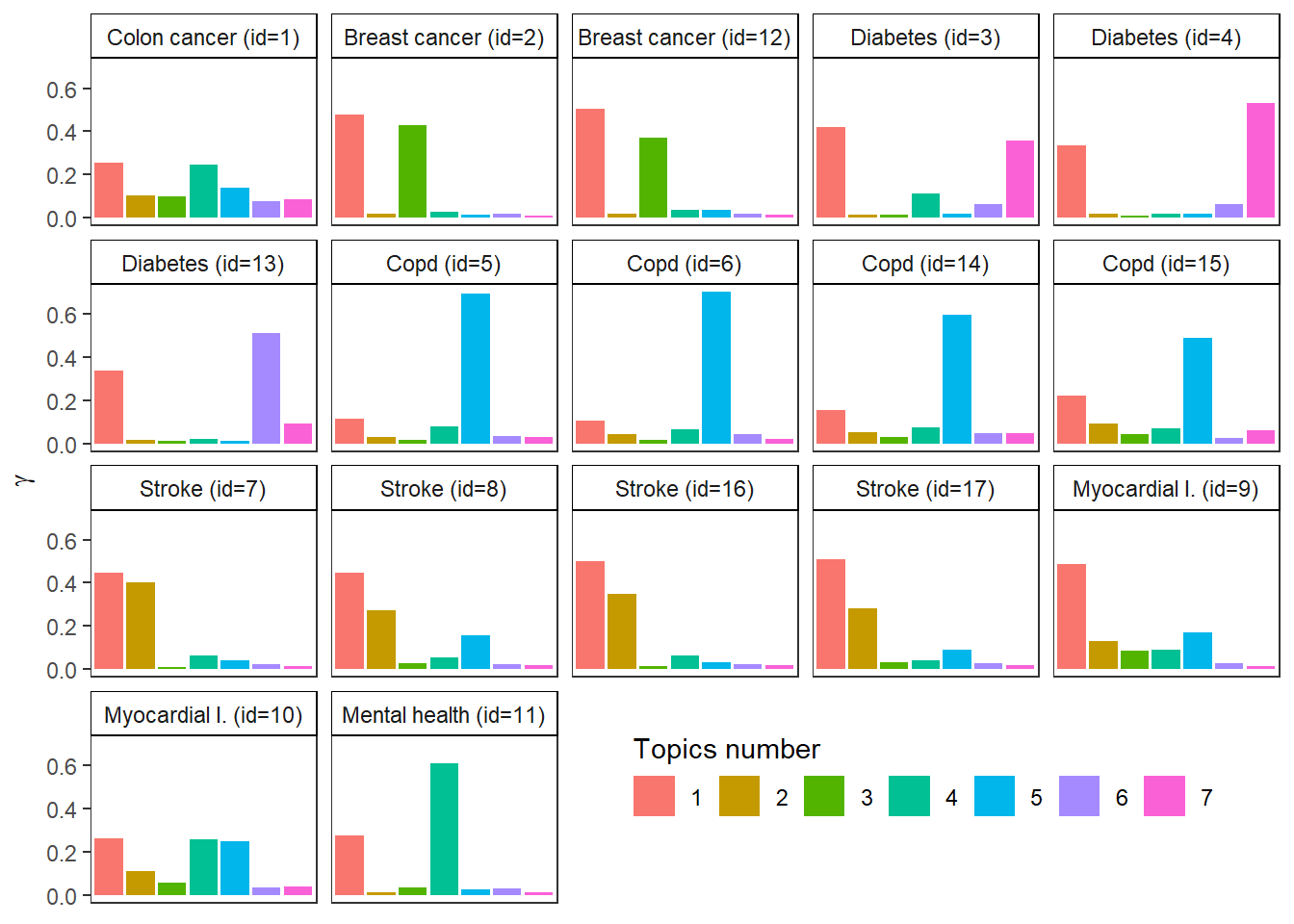


**Supplementary material. Table 5:** Text segments cluster assignment by group and and cluster weights.

|  | **Cluster 1** | **Cluster 2** | **Cluster 3** | **Cluster 4** | **Cluster 5** | **Cluster 6** |  | **Cluster 7** | **Weights** | **n** |
| --- | --- | --- | --- | --- | --- | --- | --- | --- | --- | --- |
| Breast cancer, n(%) | 0 (0) | 0 (0) | 106 (54.639) | 30 (15.464) | 56 (28.866) | 1 (0.515) |  | 1 (0.515) | Cluster 1, n(%) | 4 (23.529) |
| Colon cancer, n(%) | 0 (0) | 0 (0) | 0 (0) | 3 (50) | 2 (33.333) | 0 (0) |  | 1 (16.667) | Cluster 2, n(%) | 6 (35.294) |
| Copd, n(%) | 12 (24) | 24 (48) | 1 (2) | 2 (4) | 9 (18) | 0 (0) |  | 2 (4) | Cluster 3, n(%) | 11 (64.706) |
| Diabetes, n(%) | 0 (0) | 0 (0) | 9 (2.687) | 26 (7.761) | 16 (4.776) | 132 (39.403) |  | 152 (45.373) | Cluster 4, n(%) | 15 (88.235) |
| Mental health, n(%) | 0 (0) | 0 (0) | 2 (5) | 3 (7.5) | 24 (60) | 5 (12.5) |  | 6 (15) | Cluster 5, n(%) | 17 (100) |
| Myocardial I., n(%) | 0 (0) | 1 (3.571) | 2 (7.143) | 13 (46.429) | 10 (35.714) | 0 (0) |  | 2 (7.143) | Cluster 6, n(%) | 7 (41.176) |
| Stroke, n(%) | 0 (0) | 1 (0.415) | 16 (6.639) | 139 (57.676) | 68 (28.216) | 3 (1.245) |  | 14 (5.809) | Cluster 7, n(%) | 11 (64.706) |

Data expressed with absolute and relative values (%).

**Supplementary material. Table 6:** Sentiment and polarity post hoc tests ^a^p values.

|  | **NCR dictionary sentiments** | **NCR dictionary emotions** | **Afinn dictionary sentiments** | **Afinn dictionary scores** | **Bing dictionary sentiments** | **Polarity phrases score** |
| --- | --- | --- | --- | --- | --- | --- |
| Breast cancer : Colon cancer | >0.999 | >0.999 | 0.227 | >0.999 | >0.999 | >0.999 |
| Breast cancer : Copd | >0.999 | 0.024 | >0.999 | <0.001 | >0.999 | >0.999 |
| Breast cancer : Diabetes | <0.001 | <0.001 | <0.001 | <0.001 | <0.001 | <0.001 |
| Breast cancer : Mental health | >0.999 | 0.032 | 0.546 | <0.001 | >0.999 | >0.999 |
| Breast cancer : Myocardial infarction | <0.001 | 0.001 | 0.078 | <0.001 | 0.304 | >0.999 |
| Breast cancer : Stroke | <0.001 | <0.001 | <0.001 | <0.001 | <0.001 | <0.001 |
| Colon cancer : Copd | >0.999 | >0.999 | >0.999 | >0.999 | >0.999 | >0.999 |
| Colon cancer : Diabetes | 0.011 | 0.036 | <0.001 | 0.002 | 0.001 | 0.837 |
| Colon cancer : Mental health | >0.999 | >0.999 | 0.024 | 0.678 | >0.999 | >0.999 |
| Colon cancer : Myocardial infarction | 0.456 | >0.999 | >0.999 | >0.999 | >0.999 | >0.999 |
| Colon cancer : Stroke | 0.708 | 0.218 | 0.001 | 0.077 | 0.068 | >0.999 |
| Copd : Diabetes | <0.001 | <0.001 | <0.001 | <0.001 | <0.001 | <0.001 |
| Copd : Mental health | >0.999 | 0.003 | 0.024 | <0.001 | >0.999 | >0.999 |
| Copd : Myocardial infarction | <0.001 | <0.001 | >0.999 | 0.001 | >0.999 | >0.999 |
| Copd : Stroke | <0.001 | 0.01 | <0.001 | <0.001 | <0.001 | 0.331 |
| Diabetes : Mental health | <0.001 | <0.001 | 0.028 | <0.001 | <0.001 | >0.999 |
| Diabetes : Myocardial infarction | <0.001 | <0.001 | <0.001 | <0.001 | <0.001 | 0.052 |
| Diabetes : Stroke | <0.001 | <0.001 | 0.235 | 0.418 | <0.001 | <0.001 |
| Mental health : Myocardial infarction | <0.001 | <0.001 | 0.001 | 0.144 | >0.999 | >0.999 |
| Mental health : Stroke | <0.001 | <0.001 | >0.999 | <0.001 | <0.001 | >0.999 |
| Myocardial infarction : Stroke | <0.001 | <0.001 | <0.001 | <0.001 | <0.001 | 0.849 |

^a^significant if p<0.05 (shown in red).
